# Adolescent Parenting: Global Perspective, Local Action

**DOI:** 10.5334/aogh.2477

**Published:** 2019-04-25

**Authors:** Linda Olszewski, Angela Diaz

**Affiliations:** 1 The McShane Center for Psychological Services, Department of Psychology, Dyson College, Pace University, New York, NY, US; 2 Department of Environmental Medicine and Public Health, Icahn School of Medicine at Mount Sinai, Manhattan, NY, US; 3 Department of Psychiatry, Icahn School of Medicine at Mount Sinai, Manhattan, NY, US

## Adolescent Parenting

In recent decades, a significant decrease in the rates of teen pregnancy has been observed worldwide. Though the decline is significant, teen pregnancy continues to account for more than 10% of births globally [1]. Furthermore, the vast majority of these births to adolescent parents occur in nations of low or middle income [2]. Sub-Saharan Africa and Mexico are found to have the highest rates of adolescent births. Amongst wealthier nations, the United States has the highest rates of live births by teen mothers [1,3].

Teen pregnancy rates are influenced by a trifecta of social, cultural, and economic circumstances and rates may vary widely across socio-economic groups within countries.

Regardless of cultural expectations there are many implications of becoming a teen parent. This paper seeks to review adolescent pregnancy and parenting from a global perspective and highlight the importance of an interdisciplinary approach when addressing teen parenting and its manifestations and predictors.

## Economic Challenges

Economic challenges can act as both a predictor and an outcome of teen pregnancy globally. Findings indicate that teen pregnancy is a result of economically challenged conditions, and can lead to poor outcomes for both teen parents and their offspring [1,4]. Early parenting during the teen years, often creates obstacles to both educational and employment opportunities [1,3,4]. Unfortunately, becoming a parent during adolescence contributes to the cycle of poverty, and has a significant impact on elevated rates of both maternal and child deaths, as well as poor health in many countries regardless of economic levels [2]. Nationally, these circumstances will affect local economy (welfare programs) and thus on a larger scale global economy as well.

## Health Risks

Aside from contributing to and maintaining economic disparities, teen mothers also face many adverse health outcomes including greater risk of death [2,3]. Globally, studies have correlated adolescent pregnancy with several negative health complications such as anemia, chronic hypertension during pregnancy, inadequate weight gain during pregnancy, and STD’s [5]. Babies born to teen mothers are more likely to be preterm and underweight [5,6,7,8,9].

## Negative Parenting

To compound matters, not only do adolescent mothers also face greater difficulties engaging in effective parenting behaviors, but Smith, Chiappone, & Wilson (2017) [10] found that adolescent mothers often employed a negative parenting style. It is known that negative parenting behaviors can result in poor academic outcomes for children and are correlated with higher rates of trauma in children. Additionally, there is a growing body of literature demonstrating that adolescents who experience a negative relationship with their caregivers are more likely to engage in high risk behaviors including sex and thus are more likely to become teen parents themselves [11]. Negative parenting behaviors can be both predictors and outcomes of teen parenting highlighting the importance of parent education and dyadic work when working with a teen parent.

## Trauma

Studies have found that mothers who had experienced multiple adverse childhood experiences (ACES), such as violence, were more likely to have an unwanted pregnancy [12]. It is well established that ACES increase the risks for poor mental health and physiological effects [12,13], emphasizing the need for teen parents to receive mental health services to adequately meet their needs.

## Public Sector Costs

The cost of adolescent births to the public sector is significant [14]. The reduction in the adolescent birthrate from 1994–2010 in the United States has resulted in taxpayer savings of 12 billion dollars over that period [15]. New York has one of the highest teen pregnancy rates in the United States [4]. The cost to the state is approximately 340 million dollars annually [15]. Though rates have declined, the Bronx continues to maintain of the highest rates of teen pregnancy in New York City, with 29.4 out of 1000 births [16].

## Integrated Care Model: Medical Homes

Due to the economic challenges, health risks, negative parenting patterns, high incidence of trauma and significant cost to the public sector [14], it is important to approach teen parents from a supportive and multifaceted manner that can be successfully met by a medical home.

Poverty playing a central role as risk-factor for adolescent pregnancy highlights the importance of providing free services to this population. Ultimately, free services can be re-conceptualized as a cost-saving strategy, as cost is typically a major financial barrier to receiving preventative care for this population. More importantly, the changing demographic of the United States requires a culturally responsive and integrated approach. Adding to the complexity of care are the high rates of trauma experienced by adolescent mothers and negative parenting styles employed. The complexity of care emphasizes the intersection of mental health and medical professions. The medical, mental health and social complexities underline the importance and difficulty of providing high quality, culturally competent, integrated care for these at risk dyads.

A team of medical doctors, psychologists, social workers, health educators, dentists, and nutritionists working together leads to high-quality care that would best serve the needs of those at risk, and should contribute to meet most needs simultaneously to help remove all barriers to care. Working on improving physical and mental health, providing preventive services as part of a cohesive team effort, should increase the likelihood that high-risk dyads will receive the full array of services they need to achieve optimal health as well as prevent subsequent pregnancies.

In an effort to think globally and act locally, the Teen Parent Program at the Mount Sinai Adolescent Health Center, an innovative program, acts as model for all programs and provides free, comprehensive, integrated, medical and mental health services to a population of teen mothers and their children. Focusing on health and preventative services, the program also incorporates psychological, social work, health education, dental, legal, nutrition, optical, and sexual and reproductive services, to care. The aim is to support adolescent mothers between the ages of 10–24 and their children who are warmly welcomed into care by a team of health professionals. The team approach results in an enhanced ability to provide integrated care using a family-based model in an efficient and effective manner by enabling warm hand-offs between disciplines. Conceptualizing and prioritizing care through a hierarchy of needs, the goal is to first meet clients’ basic needs, ensuring health, housing and food stability, and following the children developmentally.

The health team is active in providing preventative care such as contraception to prevent repeat pregnancy and helps families adhere to their children’s vaccination schedule. In addition, the medical team incorporates dental risk assessments, psycho-education on the importance of dental care, and fluoride treatments into their comprehensive physical examinations; dental referrals are also made during these appointments as needed; lastly mental health referrals to social work and psychology are also provided by physicians, but decided as a team prior to meeting the dyad by the multi-disciplinary teem in a pre-clinic huddle. This medical referral acts as a way to legitimize and de-stigmatize supportive services, such as mental health, in the eyes of the clients thereby removing disparities in access to mental health providers.

The adolescent parents in the population serviced by the MSAHC, predominantly minorities, have preexisting stigmas about mental health services which acts as a significant barrier to seeking mental health care and often results in the adolescents being wary of mental health services. Mental health has been identified as central for overall health and well-being across the life span [17]. With the encouragement of trusted medical providers, parents follow a regimen of psychological and developmental screeners, and meet with social workers to complete a psychosocial assessment in order to gather information (e.g. food and housing stability, historical trauma, mental health status, psychosocial stressors). This aids the mental health team to best meet the families’ needs, by helping them acquire necessary services (e.g. Supplemental Nutrition Assistance Program [SNAP], Women, Infants and Children [WIC] benefits), serving both mothers and fathers, helping them return to school, and providing the necessary psychological interventions (eg. dyadic therapy, individual therapy, parenting interventions) to help guide the young families off the path to poverty.

One of the ongoing challenges to provide care is the inherent distrust in doctors, psychologists, social workers, who are non-family members. This highlights the importance of teens engaging in care with a trusted childcare provider in order to promote health as well as helping adolescents navigate the subsidized childcare system, provide effective referrals to other services, and building trust, which may be crucial to them as they return to school or work.

Using a trauma-informed model has been quite relevant to effective care and successful outcomes at the MSAHC. The psychology team has incorporated the Adverse Childhood Experiences measure [12] in order to build a complete picture of the parent history. Using this knowledge and being cognizant of the obstacles of engagement the team approaches mothers from all perspectives. While educating parents on parenting practices is important for these mother-child pairs, the literature demonstrates that supporting the dyad through improving *interactions* between parent and child is more effective at making long-term changes [18]. With this knowledge, the psychology team has organized the Child and Parent Support (CaPS) program to better support interactions using attachment literature as a guide [19,20,21,22,23].

Summing up, the implementation of a medical home model brings significant promise to the successful management of teen pregnancies and parenting. Our work at the MSAHC offers an inspired model that may have global applications that take into account regional, cultural and linguistic differences.

## Abbreviations

Mount Sinai Adolescent Health Center (MSAHC).
